# Comparing the Squatting Position and Traditional Sitting Position for Ease of Spinal Needle Placement: A Randomized Clinical Trial

**DOI:** 10.5812/aapm.13969

**Published:** 2014-04-05

**Authors:** Sussan Soltani Mohammadi, Marzieh Hassani, Seyed Mojtaba Marashi

**Affiliations:** 1 Department of Anesthesiology, Dr. Shariati Hospital, Tehran University of Medical Sciences, Tehran, Iran

**Keywords:** Squatting Position, Anesthesia, Spinal, Spinal Needle, Traditional Sitting Position

## Abstract

**Background:**

previous evidences suggested that traditional sitting position (flexion of knees approximately 90°, and adduction of hips while feet rest on a stool) and hamstring stretch position (sitting position with maximum extension of knees, adduction of hips, and forward bending) both reversed the lumbar lordosis and the number of spinal needle-bone contacts were identical when placing patients in these positions for neuraxial block.

**Objectives:**

In this study, we suggested that squatting position reverses the lumbar lordosis and reduces the number of spinal needle bone contacts better than a traditional sitting position.

**Patients and Methods:**

Two hundred and thirty six patients ASA (American Society of Anesthesiologist) class I or II aged 18 to 75 years scheduled for elective surgeries under elective spinal anesthesia were randomized into two groups. We compared the traditional sitting and squatting positions. Our primary endpoint was the number of spinal needle-bone contacts, and secondary endpoint was ease of needle insertion or space identification.

**Results:**

The total number of spinal needle bone contact was statistically lower in the squatting position compared to traditional sitting position group (222 versus 230 respectively, P = 0.01). Insertion of needle was easy in 97 (87%) and 94 (84%) of patients and difficult in 20 (18%) and 17 (15%) of patients in traditional sitting and squatting positions, respectively (P = 0.59 and P = 0.12). Needle insertion was not impossible in any patients.

**Conclusions:**

In squatting position the number of spinal needle-bone contacts was lower compared to the traditional sitting position, nonetheless ease of needle insertion or space identification was the same in the both groups.

## 1. Background

Identification of spinal space and reducing the number of spinal needle-bone contacts could be facilitated by reducing lumbar lordosis during initiation of neuraxial block. In a study by Tashayod et al. a kind of modified sitting position with maximum extension of knees, adduction of hips, and forward bending (hamstring stretch position) was described more effective in reducing lordosis of lumbar spine and making spinal puncture easier. Even moderate passive knee extension of a patient in sitting position can increase hamstring tension, tilting the pelvis, and reduce lumbar lordosis (1, 2). In a randomized trial by Fisher et al. 205 patients in traditional sitting position were compared with 201 patients in hamstring stretch position regarding the number of spinal needle bone contact during epidural labor analgesia. The number of needle-bone contacts were the same in the both groups (3).

Traditionally, patients are placed with their back flexed in either lateral or sitting position to perform subarachnoid block for spinal anesthesia. Flexed back for subarachnoid block is considered mandatory due to widening of inter spinous space in this state (4, 5). Although flexed back is considered mandatory for subarachnoid block, it may be uncomfortable for some patients to assume a flexed posture. In this regard, Biswas et al. conducted a study to find out the degree of successful procedure and patient’s preference when spinal block was performed on suboptimal lumbar spine flexion. They found that in both lateral or seated positions, the success rates were 100% and 95% in patients with flexed or straight lumbar spine position (6). Reducing lumbar lordosis can be also induced by squatting which increases hamstring tension. In this position, the patient squats while his or her buttock and plantar surfaces of the feet are supported by the operating table and patient hugs his or her knees (personal experience, unpublished observation).

## 2. Objectives

We hypothesized that squatting position would reduce spinal needle bone contact compared to traditional sitting position by reducing lordosis of lumbar spine and improving needle insertion or space identification. Our primary goal was to minimize needle bone contact and the second was ease of needle insertion/space identification.

## 3. Patients and Methods

This randomized clinical trial was performed in Dr. Shariati Hospital of Tehran University of Medical Sciences from November 2011 to March 2012. The study protocol conformed to the ethical guidelines of the 1989 Declaration of Helsinki.

Ethical approval for this study was provided by the Ethical Committee of Tehran University of Medical Sciences. During the pre-anesthetic meeting, patients were instructed about the proposed posture for the procedure according to the randomization. A written informed consent was obtained individually before the operation.

Two hundred fifty six patients, ASA class I or II aged 18 to 75 years, scheduled for elective lower abdominal or lower extremity surgeries under spinal anesthesia were enrolled in the study. Randomization was based on computer-generated codes and was concealed in envelopes, opened by the block performer before the spinal puncture. Exclusion criteria included language barrier, contraindications to neuraxial block, pregnancy, morbid obesity (BMI > 40), lumbar surgical scar and obvious lumbar scoliosis. Patients were premedicated with oral chlordiazepoxide 10 mg the night before and on the morning of operation.

All patients had an intravenous (IV) infusion placed and were given isotonic saline 3 mL/kg before spinal anesthesia. Standard monitoring was used during spinal anesthesia. All spinal blocks were performed by two anesthesiologists who had experience of more than 400 spinal procedures in the traditional sitting position and random spinal anesthesia using squatting position. The block performer sat on a stool at a level to keep the interspinous space at the level of his or her eyes.

The block performers used a 25-gauge needle with a length of 3.8 cm, for local anesthetic injection followed by a 25-gauge Quincke needle by midline approach at L2-L3 or L3-L4 interspace. An anesthesiology resident recorded weight, height and the surface landmarks graded by block performer as: easy, difficult, or impossible to palpate the lumbar spinous processes, while the patient sat in a study group position according to the allocation.

**Figure 1.**
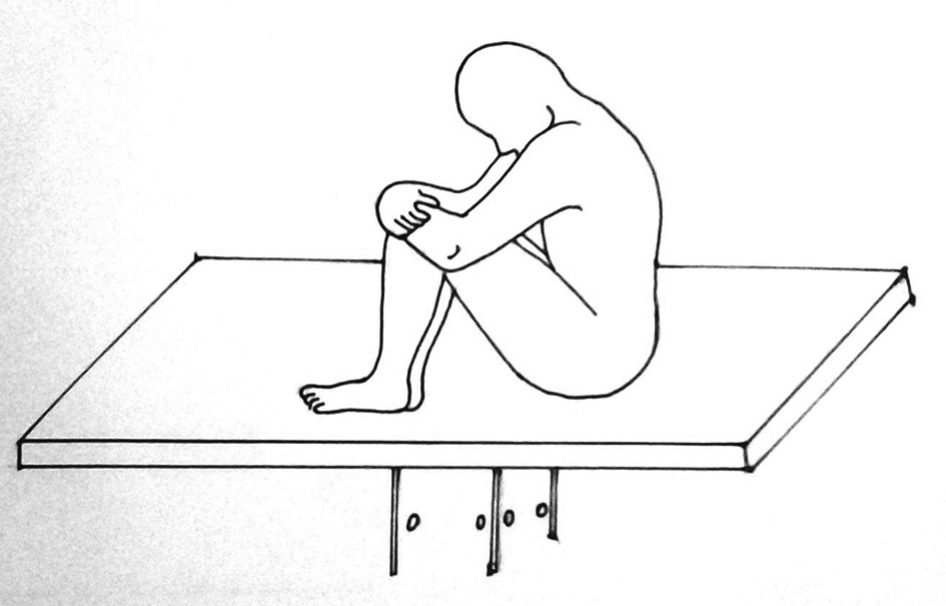
Squatting Position Patients sit with their lower extremities fully flexed at hip and knee joints, while hugging their knees and both buttock and plantar surfaces of the feet supported by the bed and forward lean of the torso.

For traditional sitting position, patients flexed their knees approximately 90° and adducted hips and put their feet on a stool; height of the bed was adjusted to provide maximum hip and lumbar flexion. In squatting position, patients sat with their lower extremities fully flexed at hip and knee joints while hugging their knees and both buttock and plantar surfaces of the feet were supported by the bed and forward bending (Figure 1). In both groups, “maximum” was the greatest amount that patient could tolerate.

The spinal procedures were performed to improve needle insertion or space identification and minimize spinal needle-bone contacts. A spinal needle-bone contact was defined as spinal needle contact against bone which prevented further passage. All spinal needle-bone contacts were also recorded. After inserting the needle and withdrawing the stylet, appearance of cerebrospinal fluid (CSF) in the hub of the needle was evaluated. The study was complete whenever the subarachnoid space was confirmed by the aspiration of free flow of CSF. When there was either no CSF in the needle hub or there was scanty of CSF with poor flow, the needle was rotated clockwise 90 degrees and waited for 5 seconds. The sequence of rotation continued for another three-quadrant rotation of 90 degrees and waited for 5 seconds between each rotation. Despite this maneuver, if there was absence of CSF or its free flow, the needle was further advanced approximately by 2 mm. The block performer was not allowed to perform a new puncture site and was restricted to pull back the needle just to the subcutaneous tissue. When bone was encountered during any of above mentioned-attempts, the needle was withdrawn just below the skin level followed by reinsertion with a more cephalad angulation. If more than five spinal needle-bone contacts occurred, the case was recorded as a failure of the position and the study was stopped.

In a pilot study of 20 patients having spinal anesthesia by 25-guage Quincke needle in traditional sitting position, 40% of them experienced no needle bone contacts (unpublished observation). Presuming that squatting position would increase this proportion to 60%; one would need to enroll 111 patients in each group for the results to be statistically significant at a power of 85% with a level of confidence of 5%. If more than 10% of sample size were excluded due to failure, the study would stopped. Data was analyzed by using SPSS version 11.5 (SPSS Inc., Chicago, IL). Normal distribution of data was checked by Kolmogorov Smirnov test. Independent sample’s t-test, ANOVA, Chi-square and Fisher’s exact tests were used when appropriate. P ≤ 0.05 was considered statistically significant.

## 4. Results

**Figure 2.**
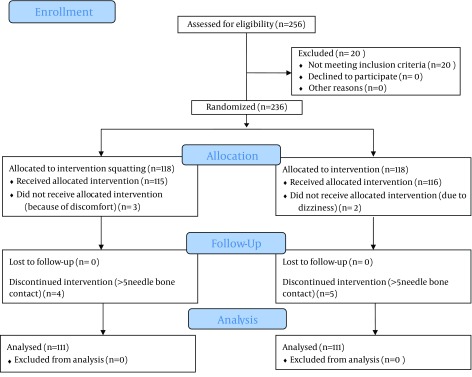
Consort Flow Diagram

In total, 236 of 256 patients scheduled consecutively for spinal anesthesia, were randomized into two equal groups (n = 118), and 20 were excluded due to fulfilling the exclusion criteria. Three patients of squatting group discontinued the position because of discomfort and two patients withdrew from traditional sitting position because of dizziness. Four of 115 squatting patients and five of 116 sitting position patients were considered as “failures” (> 5 needle-bone contacts) (P = 0.63) (Figure 2).

Demographic data were not statistically different between the study groups (Table 1, P > 0.05).

**Figure 3.**
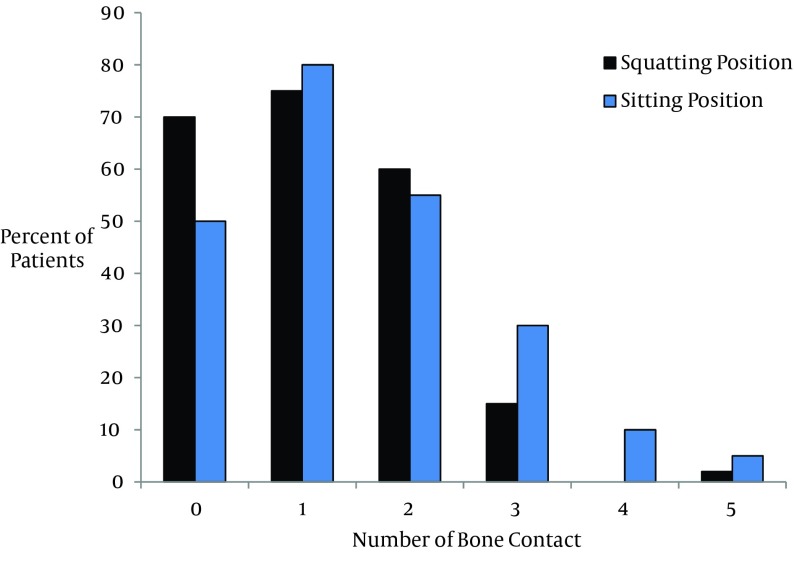
Comparing Number of Bone Contacts Between the Study Groups

Insertion of needle was easy in 97 (87%) and 94 (84%) of patients and difficult in 20 (18%) and 17 (15%) of patients in traditional sitting and squatting positions, respectively (P = 0.59 and P = 0.12). Insertion of needle was not impossible in any patients. The total number of bone contact was statistically lower in squatting position compared to traditional sitting position group (222 versus 230 respectively, P = 0.01, Figure 3).

**Table 1.** Comparing Demographic Data Between the Study Groups ^a,b,c^

Variable	Squatting (n = 111)	Sitting (n = 111)
**Age, y**	40 ± 0.8	40 ± 13.8
**Sex (Male/Female)**	60/51	62/49
**Weight, kg**	68.8 ± 10.6	63.8 ± 15.1
**Height, cm**	165.9 ± 9.3	166.1 ± 11.5
**BMI**	25.7 ± 2.8	26.1 ± 1.93

^a^ Abbreviations: BMI, body mass index.

^b^ Data are presented as mean ± SD.

^c^ P > 0.05.

## 5. Discussion

This study demonstrated the same findings between the squatting and traditional sitting positions regarding ease of spinal needle insertion or space identification, nonetheless the number of needle bone contact was lower in the squatting group. Although, squatting position decreases lumbar lordosis, space identification or needle insertion was not different with traditional sitting position. This may be due to inducing tension in the supraspinous ligament. In a study by Fisher et al. on 406 patients requested for epidural analgesia, number of needle bone contact was equal in the both groups (traditional sitting and hamstring stretch position), which was different to our patients’ position (3). Although flexed back is considered mandatory for subarachnoid block, it may be uncomfortable for some patients to assume a flexed posture. Biswas et al. conducted a study on 160 patients undergoing spinal anesthesia with suboptimal flexion of lumbar spine to find the success rate and patient’s preference of the position (6).

The blocks were performed in the lateral or seated position with the back either flexed or straight. Their patients were divided into four groups: lateral with back straight (LS) or flexed (LF) and seated with back straight (SS) or flexed (SF). Their primary outcome was correct spinal needle placement. Number of attempts, needle redirections and patients' preferred posture were compared with the outcome in four groups. They found that for both positions, the success rates were 95% and 100% in straight or flexed lumbar spine, respectively (P = 0.81). In the lateral position, more patients of the LF group (40) than those of the LS group (32) had successful placement of spinal needle (P = 0.03). Thirty-four and 21 patients in the SS and SF groups, required cephalad redirections of the needle (P = 0.003). Most patients preferred the straight lumbar spine position (69.7-88%). They concluded that with a higher preference for the straight lumbar spine by patients, the overall success rate of correct spinal needle placement was comparable among the groups who had straight flexed posture for subarachnoid block.

Although the technique of position in Biswas et al. study was different to us, the overall success rate was not statistically different between their study groups which correlated with our study. However, in lateral position the success rate of needle placement at first attempt in flexed group was more than straight group which could emphasize on the role of flexion on space identification (6-9). Our study limitations were lack of blinding and inability of morbid obese and pregnant population to keep in the squatting position. On the other hand, the hamstring stretch position actually increased lumbar flexion, but by inducing tension in the supraspinous ligament that could obliterate the inter spinous depressions. We suggest further studies to compare three squatting, traditional sitting and hamstring stretch positions with larger sample size to identify ease of palpation after placing the patient in the study position.
